# Author Correction: p21-activated kinase 4 suppresses fatty acid β-oxidation and ketogenesis by phosphorylating NCoR1

**DOI:** 10.1038/s41467-023-42957-1

**Published:** 2023-11-02

**Authors:** Min Yan Shi, Hwang Chan Yu, Chang Yeob Han, In Hyuk Bang, Ho Sung Park, Kyu Yun Jang, Sangkyu Lee, Jeong Bum Son, Nam Doo Kim, Byung-Hyun Park, Eun Ju Bae

**Affiliations:** 1 Department of Biochemistry and Molecular Biology, Jeonbuk National University Medical School, Jeonju, 54896 Republic of Korea; 2 School of Pharmacy, Jeonbuk National University, Jeonju, 54896 Republic of Korea; 3 Department of Pathology, Jeonbuk National University Medical School, Jeonju, 54896 Republic of Korea; 4 School of Pharmacy, Sungkyunkwan University, Suwon, 16419 Republic of Korea; 5 VORONOI BIO Inc., Incheon, 21984 Republic of Korea

**Keywords:** Hepatocytes, Phosphorylation, Kinases

Correction to: *Nature Communications* 10.1038/s41467-023-40597-z, published online 17 August 2023

The original version of this Article contained incorrect images for the microscopy in Figure 6i and western blots in 6j. In figure 6i, the H&E staining for ND50 was inadvertently duplicated from the ND25 panel. Figure 6j included incorrect representative western blot images for NCoR1 and HSP90. The correct images were present during the review process and the error was introduced during the final editing of the manuscript.

The correct image for Figure 6i is:


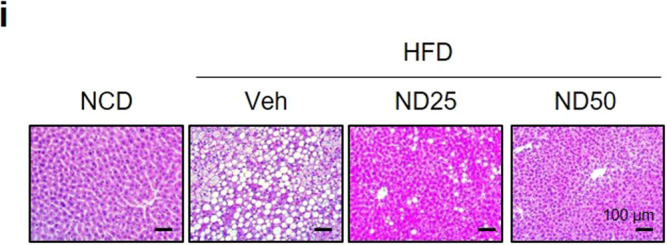


The correct image for Figure 6j is:


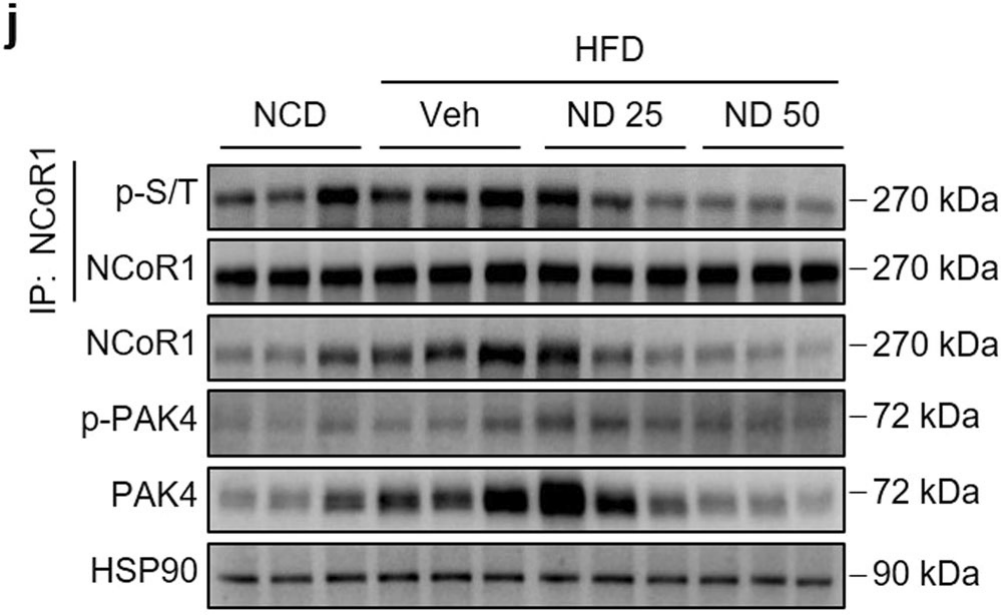


These errors have been corrected in both the PDF and HTML versions of the Article.

