# Supplementary material for: p21-activated kinase 4 suppresses fatty acid β-oxidation and ketogenesis by phosphorylating NCoR1
Source: Nat Commun. 2023 Aug 17;14:4987. doi: 10.1038/s41467-023-40597-z (PMC10435519; doi:10.1038/s41467-023-40597-z)
Supplement: Supplementary file 1 — Supplementary Information [file 41467_2023_40597_MOESM1_ESM.pdf]

p21-activated kinase 4 suppresses fatty acid β-oxidation and ketogenesis by phosphorylating NCoR1

Min Yan Shi[1], Hwang Chan Yu[1], Chang Yeob Han[2], In Hyuk Bang[1], Ho Sung Park[3], Kyu Yun Jang[3], Sangkyu Lee[4], Jeong Bum Son[5], Nam Doo Kim[5], Byung-Hyun Park[1,$], and Eun Ju Bae[2,$]

[1]Department of Biochemistry and Molecular Biology, Jeonbuk National University Medical School, Jeonju 54896, Republic of Korea

[2]School of Pharmacy, Jeonbuk National University, Jeonju 54896, Republic of Korea

[3]Department of Pathology, Jeonbuk National University Medical School, Jeonju 54896, Republic of Korea

[4]School of Pharmacy, Sungkyunkwan University, Suwon 16419, Republic of Korea

[5]VORONOI BIO Inc., Incheon 21984, Republic of Korea

Running title: PAK4 inhibition facilitates ketogenesis

Supplementary materials

1. Supplementary Figures
2. Supplementary Tables

1. Supplementary Figures

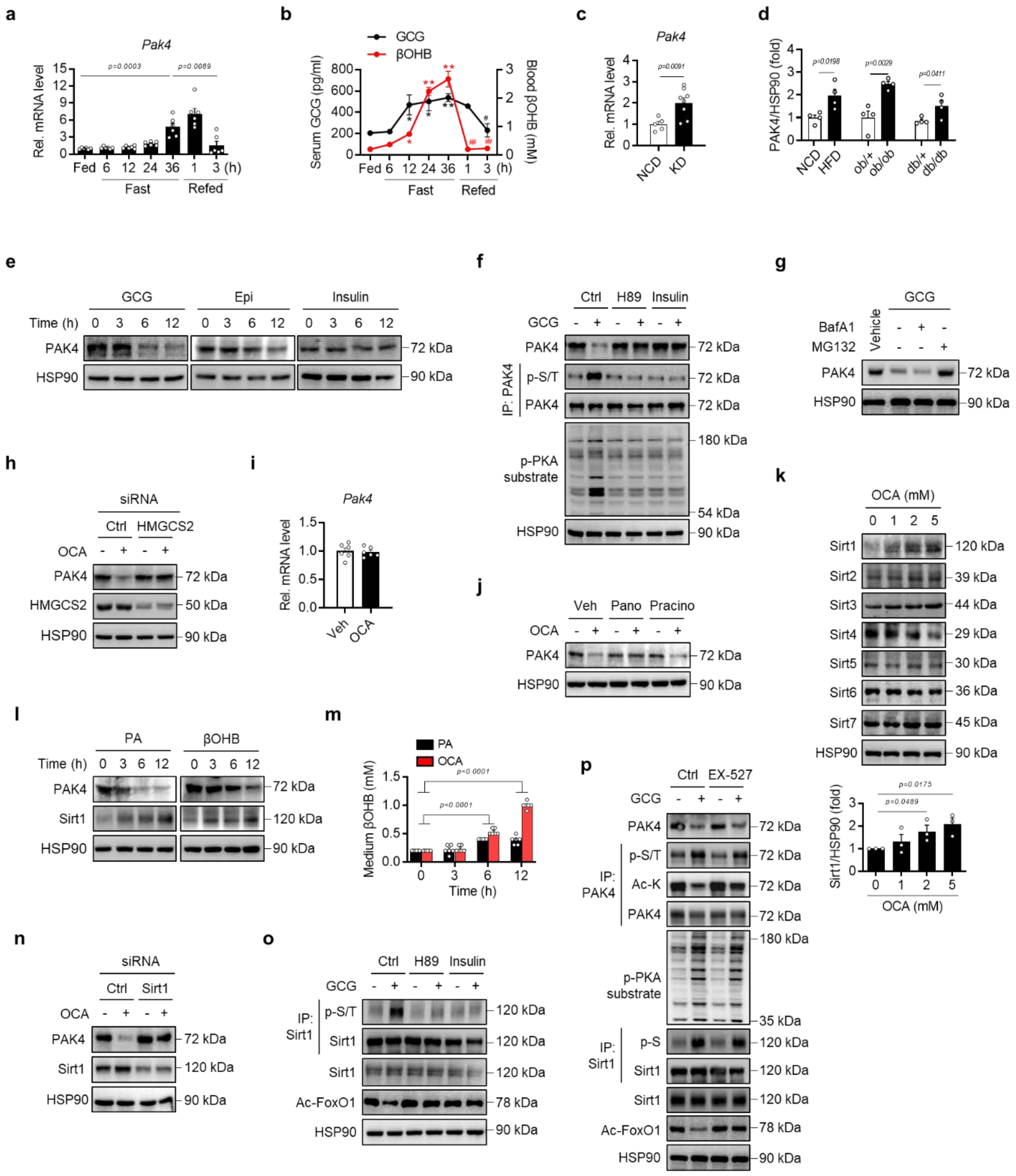

Supplementary Figure 1. Regulation of PAK4 protein and mRNA levels under various conditions (compare to Figure 1). a, **b**. Eight-week-old male C57BL/6 mice were fed either a normal chow diet (NCD) *ad libitum*, fasted for 6–36 h, or refed for 1 or 3 h following a 36 h fast. Hepatic *Pak4* mRNA levels (a, n = 6) and blood β-hydroxybutyrate (βOHB) and serum

glucagon (GCG) levels (b, *n* = 3) were analyzed. $^{*}p<0.05$ and $^{**}p<0.01$ versus fed; $^{\#}p<0.05$ and $^{\#\#}p<0.01$ versus fasted for 36 h. **c**. Eight-week-old male C57BL/6 mice were fed either a NCD or a ketogenic diet (KD) for 2 weeks, and hepatic *Pak4* mRNA levels were analyzed (*n* = 5 for NCD and *n* = 8 for KD). **d**. The protein densities in Fig. 1c were quantified (*n* = 4). **e**. Mouse primary hepatocytes were treated with glucagon (100 nM), epinephrine (Epi, 100 μM), or insulin (100 nM) for the indicated times for PAK4 immunoblotting. **f**. Primary hepatocytes were treated with glucagon (100 nM) for 12 h in the presence or absence of H89 (10 μM) or insulin (100 nM). Cell lysates were immunoblotted for indicated proteins or immunoprecipitated with anti-PAK4 antibody followed by immunoblotting with anti-p-Ser/Thr (p-S/T) antibody. **g**. Primary hepatocytes were treated with glucagon (100 nM) for 12 h with or without MG132 (2 μM) or bafilomycin A1 (BafA1, 100 nM), and PAK4 protein levels were assessed by Western blotting. **h**, **i**. Primary hepatocytes were transfected with siRNA targeting HMGCS2 (h) or left untransfected (i), followed by treatment with octanoate (OCA, 2 mM) for 12 h. Protein levels of PAK4 were analyzed by Western blotting, while mRNA levels were assessed using qPCR (*n* = 6). **j**. Primary hepatocytes were treated with octanoate (2 mM) for 12 h in the presence or absence of panobinostat (pano, 10 nM) or pracinostat (pracino, 100 nM). Protein levels of PAK4 were determined by Western blotting. **k**. Primary hepatocytes were treated with indicated concentrations of octanoate for 12 h. Protein levels of sirtuins were determined by Western blotting (*n* = 3). **l**, **m**. Primary hepatocytes were treated with palmitate (PA, 0.5 mM), βOHB (5 mM) for indicated time periods and the protein levels of PAK4 and Sirt1 were analyzed (l). βOHB release into culture medium in cells treated with palmitate or octanoate were compared (m, *n* = 6). **n**. Primary hepatocytes were transfected with siRNA targeting Sirt1 and then treated with octanoate (2 mM) for 12 h. Protein levels of PAK4 were analyzed by Western blotting. **o**, **p**. Primary hepatocytes were treated with 100 nM glucagon for 12 h with or without either H89 (10 μM), insulin (100 nM), or EX-527 (100 nM). Phosphorylation of Sirt1 and phosphorylation and acetylation of PAK4 were analyzed in

immunoprecipitates of each protein. p-PKA substrate and acetylated (Ac)-FoxO1 were measured as an indicator of PKA activation and Sirt1 deactivation, respectively. Data are presented as the mean ± SEM. One-way ANOVA followed by Dunnett's multiple comparisons test (a, b, k, m) and unpaired two-tailed *t* test (c, d, i) were conducted for statistical analyses. Source data are provided as a Source Data file.

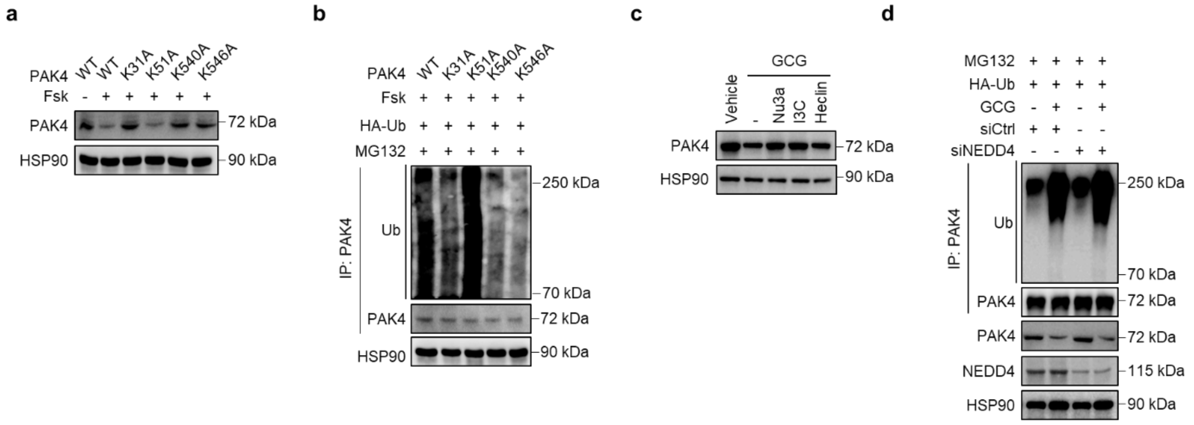

Supplementary Figure 2. PAK4 degradation *via* ubiquitin-proteasome pathway (compare to Figure 1). a, **b**. AML12 cells were transfected with wild type (WT) or lysine mutants of PAK4 (K31A, K51A, K540A, or S546A) and then treated with forskolin (Fsk, 10 μM) for 12 h to compare protein degradation and ubiquitination of PAK4. **c**. Mouse primary hepatocytes were treated with glucagon (GCG, 100 nM) for 12 h with or without nutlin-3a (Nu3a, 10 μM), indol-3-carbinol (I3C, 100 μM), and heclin (100 μM), and PAK4 protein levels were assessed by Western blotting. **d**. AML12 cells transfected with HA-Ub were treated with glucagon (100 nM) for 12 h in the presence or absence of siNEDD4. Cell lysates were immunoblotted for indicated proteins or immunoprecipitated with anti-PAK4 antibody followed by immunoblotting with anti-ubiquitin (Ub) antibody. Source data are provided as a Source Data file.

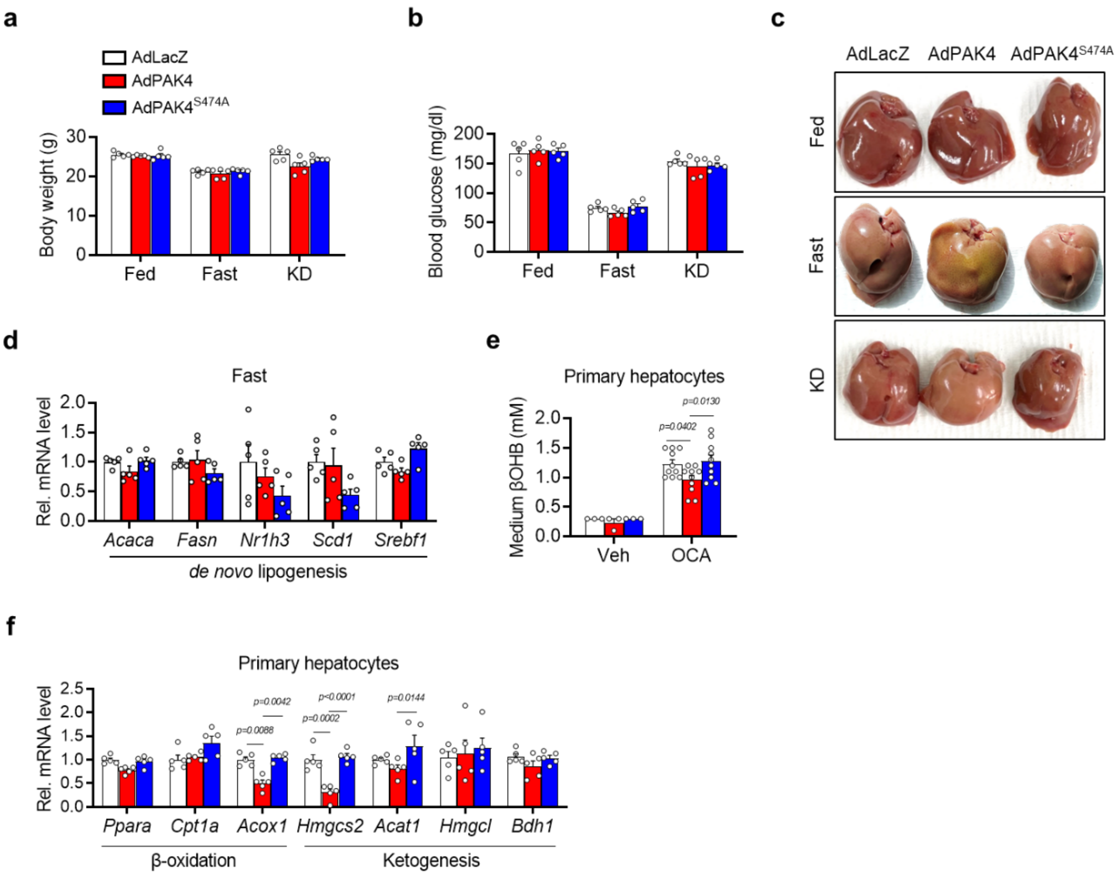

Supplementary Figure 3. Worsening of hepatic fat accumulation by PAK4 overexpression (compare to Figure 2). a, **b**. Body weight (a, n = 5) and blood glucose levels (b, n = 5) were determined in control and PAK4 overexpressing mice. **c**. Representative images of gross liver morphology. **d**. qPCR analyses of lipogenesis-related genes were performed on liver tissue obtained from fasted mice (n = 5). **e**, **f**. Mouse primary hepatocytes were infected with adenoviruses of PAK4 or PAK4^{S474A}, and then treated with octanoate (OCA, 2 mM) for 24 h. The release of βOHB into the culture medium (n = 3 for Veh group and n = 10 for OCA group) and qPCR analysis of fatty acid β-oxidation and ketogenesis genes (n = 5) were determined. Data are presented as the mean ± SEM. One-way ANOVA followed by Tukey's multiple comparisons test was conducted for statistical analyses (a, b, d, e, f). Source data are provided as a Source Data file.

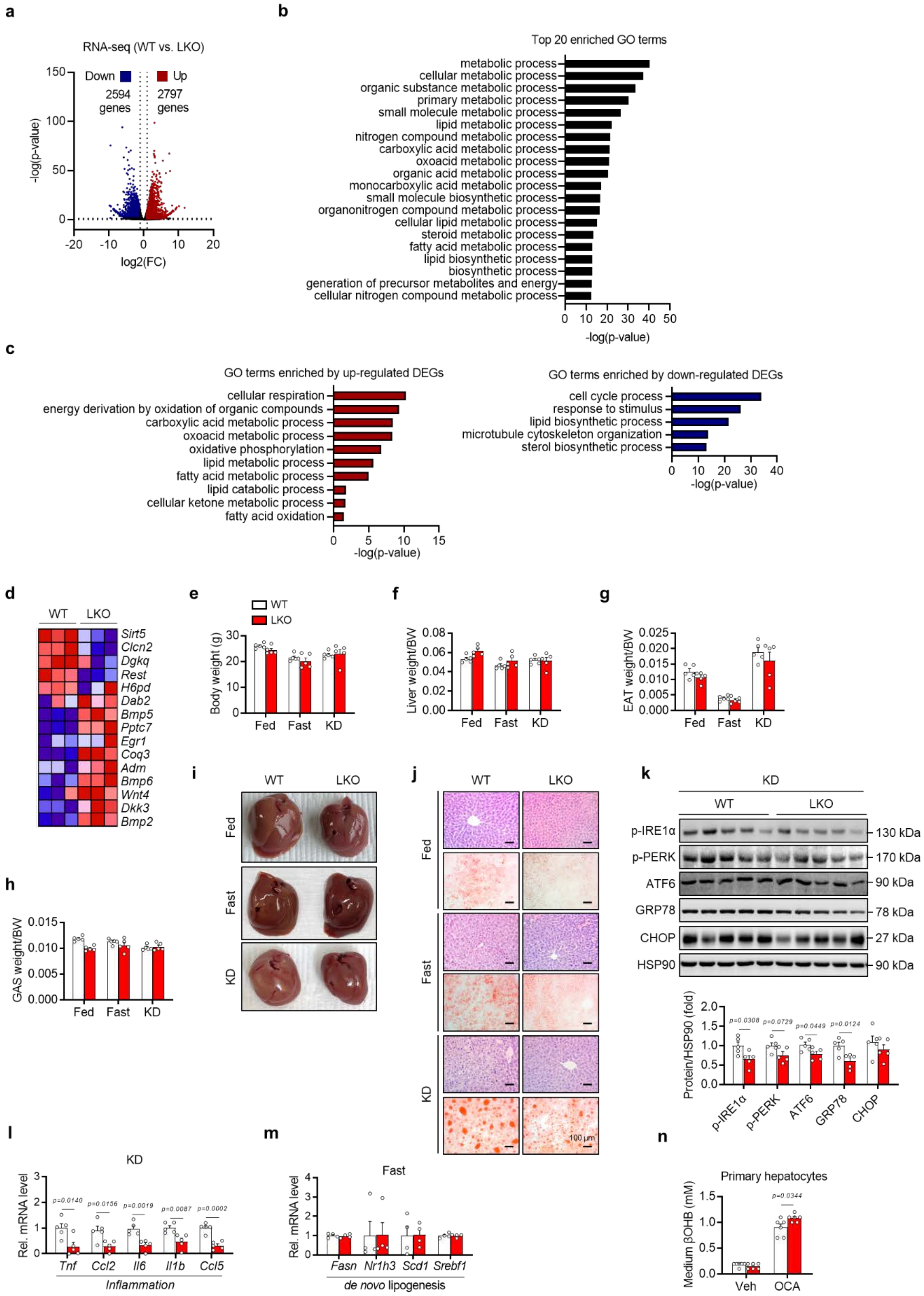
a
RNA-seq (WT vs. LKO)
Down
Up
2594 genes
2797 genes
-log(p-value)
log2(FC)
b
Top 20 enriched GO terms
metabolic process
cellular metabolic process
organic substance metabolic process
primary metabolic process
small molecule metabolic process
lipid metabolic process
nitrogen compound metabolic process
carboxylic acid metabolic process
oxoacid metabolic process
organic acid metabolic process
monocarboxylic acid metabolic process
small molecule biosynthetic process
organonitrogen compound metabolic process
cellular lipid metabolic process
steroid metabolic process
fatty acid metabolic process
lipid biosynthetic process
biosynthetic process
generation of precursor metabolites and energy
cellular nitrogen compound metabolic process
-log(p-value)
c
GO terms enriched by up-regulated DEGs
cellular respiration
energy derivation by oxidation of organic compounds
carboxylic acid metabolic process
oxoacid metabolic process
oxidative phosphorylation
lipid metabolic process
fatty acid metabolic process
lipid catabolic process
cellular ketone metabolic process
fatty acid oxidation
-log(p-value)
GO terms enriched by down-regulated DEGs
cell cycle process
response to stimulus
lipid biosynthetic process
microtubule cytoskeleton organization
sterol biosynthetic process
-log(p-value)
d
WT
LKO
Sirt5
Clcn2
Dgkq
Rest
H6pd
Dab2
Bmp5
Pptc7
Egr1
Coq3
Adm
Bmp6
Wnt4
Dkk3
Bmp2
e
WT
LKO
Body weight (g)
Fed
Fast
KD
f
Liver weight/BW
Fed
Fast
KD
g
EAT weight/BW
Fed
Fast
KD
h
GAS weight/BW
Fed
Fast
KD
i
WT
LKO
Fed
Fast
KD
j
WT
LKO
Fed
Fast
KD
100 μm
k
KD
WT
LKO
p-IRE1α
p-PERK
ATF6
GRP78
CHOP
HSP90
130 kDa
170 kDa
90 kDa
78 kDa
27 kDa
90 kDa
Protein/HSP90 (fold)
p=0.0308
p=0.0729
p=0.0449
p=0.0124
p-IRE1α
p-PERK
ATF6
GRP78
CHOP
l
KD
Rel. mRNA level
p=0.0140
p=0.0156
p=0.0019
p=0.0087
p=0.0002
Tnf
Ccl2
Il6
Il1b
Ccl5
Inflammation
m
Fast
Rel. mRNA level
Fasn
Nr1h3
Scd1
Srebf1
de novo lipogenesis
n
Primary hepatocytes
Medium βOHB (mM)
p=0.0344
Veh
OCA

Supplementary Figure 4. RNA sequencing analysis and metabolic profiles of *Pak4* LKO and WT mice (compare to Figure 3). Eight-week-old male *Pak4* LKO and WT mice were fasted for 24 h and liver samples were subjected to RNA-Seq analysis. **a**. Volcano plot showing differentially expressed genes (DEGs) in the liver of *Pak4* LKO and WT mice. Blue and red dots represent downregulated or upregulated DEGs, respectively, based on fold-changes >2 for genes with $p<0.05$. Each sample was analyzed in triplicate ($n = 3$). Statistical analyses were conducted using a two-sided negative binomial Wald test. **b**, **c**. Gene ontology (biological process category) analyses of the total DEGs (b) and upregulated or downregulated DEGs (c) in the liver. Statistical significance was determined using adjusted *p*-values, which were obtained by conducting a one-sided hypergeometric test and then applying multiple testing corrections using the Benjamini-Hochberg FDR method. **d**. Heatmap of DEGs assigned to the ketone biosynthetic process. **e**-**h**. Weights of the body (e, $n = 5$), liver (f, $n = 5$), epididymal adipose tissue (EAT) (g, $n = 5$), and gastrocnemius muscle (GAS) (h, $n = 5$). **i**, **j**. Gross liver morphology (i) and hematoxylin and eosin (H&E) and Oil Red O (ORO) staining of liver sections (j, scale bars, 100 µm). **k**, **l**. Western blotting was performed to detect endoplasmic reticulum stress markers (k, $n = 5$), and qPCR analysis was conducted to assess the expression of inflammatory genes (l, $n = 5$) in mouse livers under a KD. **m**. qPCR analysis for *de novo* lipogenesis genes ($n = 4$). **n**. Primary hepatocytes from *Pak4* LKO and WT mice were incubated with octanotate (OCA, 2 mM) for 24 h, and βOHB was measured in the culture supernatants ($n = 6$). Data are presented as the mean ± SEM. Unpaired two-tailed *t* test was conducted for statistical analyses (e-h, k-n). Source data are provided as a Source Data file.

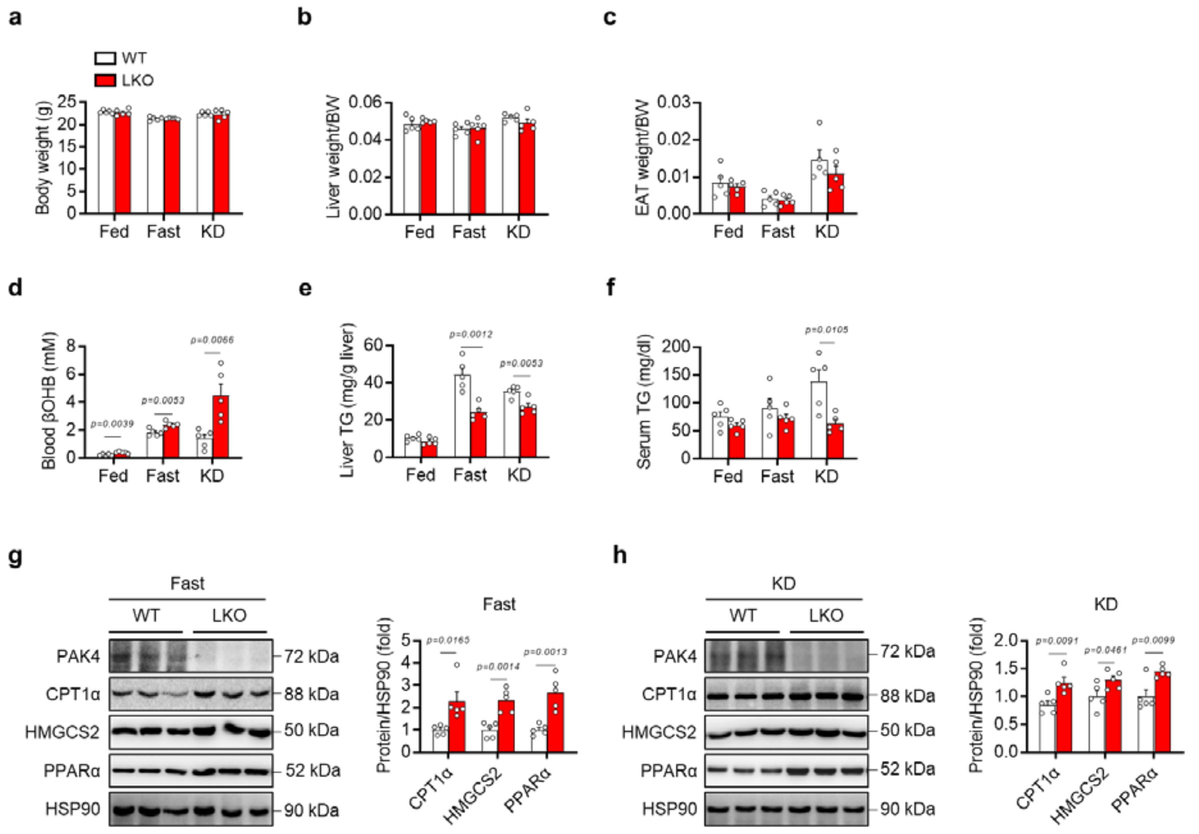

Supplementary Figure 5. Metabolic profiles of female *Pak4* LKO and WT mice after fasting or ketogenic diet feeding (compare to Figure 3). Eight-week-old female *Pak4* LKO and WT mice were fed normal chow ad libitum (Fed), fasted for 24 h (Fast), or fed a ketogenic diet (KD) for 2 weeks. **a**-**c**. Weights of the body (a), liver (b), and epididymal adipose tissue (EAT) (c). **d**-**f**. Blood levels of βOHB (d) and TG levels in the liver (e) and serum (f) were compared. **g**, **h**. Western blotting analysis of liver tissue obtained from *Pak4* LKO and WT mice after fasting (f, n = 5) or KD-feeding (h, n = 5). Data are presented as the mean ± SEM. Unpaired two-tailed t test was conducted for statistical analyses (a–h). Source data are provided as a Source Data file.

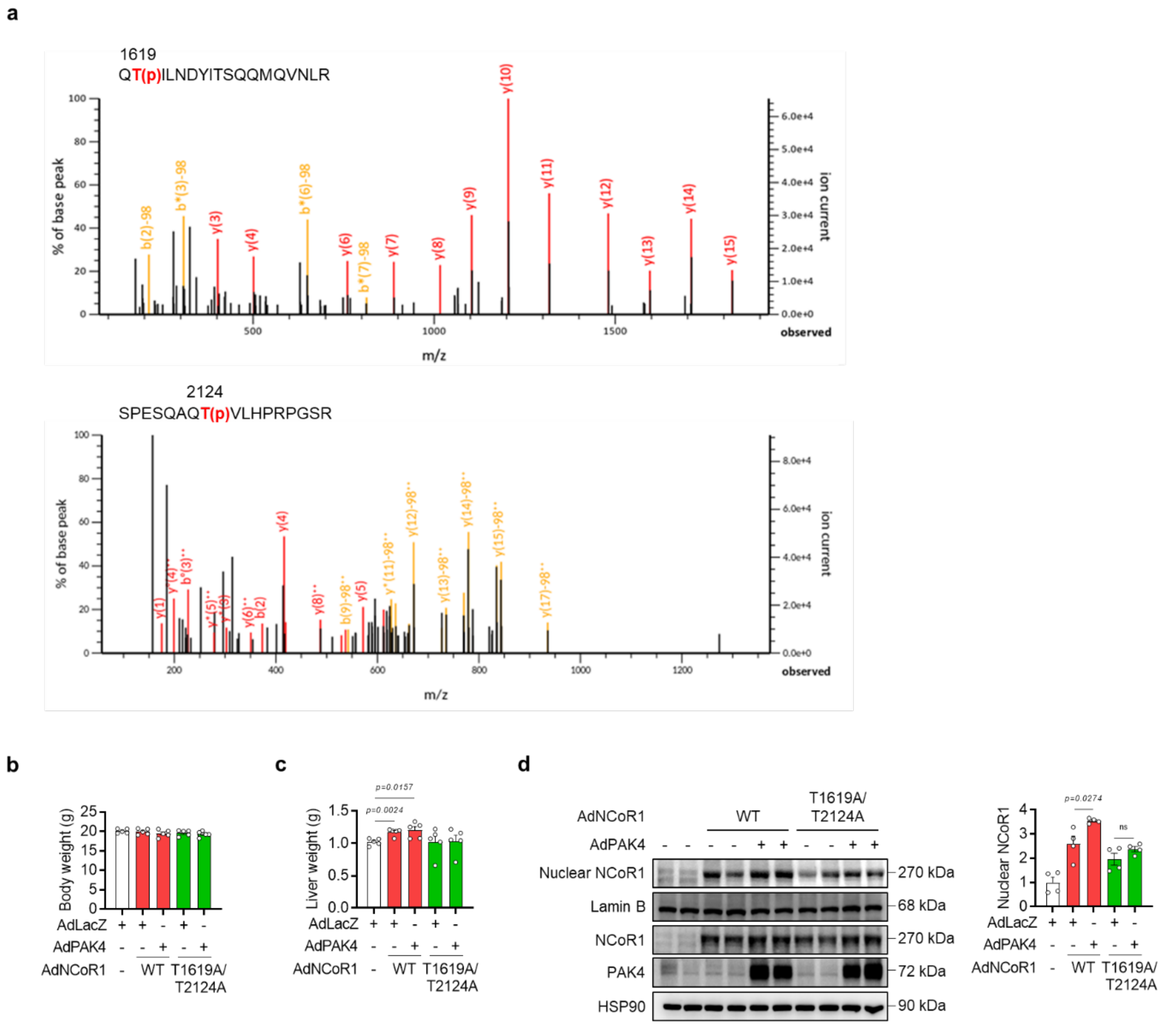

Supplementary Figure 6. LC-MS/MS analysis for NCoR1 phosphorylation and metabolic phenotypes of mice injected with PAK4 and NCoR1 adenoviruses (compare to Figure 5). **a**. Recombinant PAK4 was incubated for 30 min with two different peptides comprising T1619 or T2124 of NCoR1 in kinase assay buffer. MS/MS spectra revealing the phosphorylation of NCoR1 at T1619 (top) and T2124 (bottom) were observed. **b-d**. The experimental procedures were identical to those described in the legend to Figure 5h-j. Body weight (b, $n = 5$), liver weight (c, $n = 5$), and the levels of NCoR1 in the nuclear fraction and in the whole lysates of liver tissues (d, $n = 4$) were determined. Data are presented as the mean ± SEM. Unpaired two-tailed t test was conducted for statistical analyses (b-d). Source data are provided as a Source Data file.

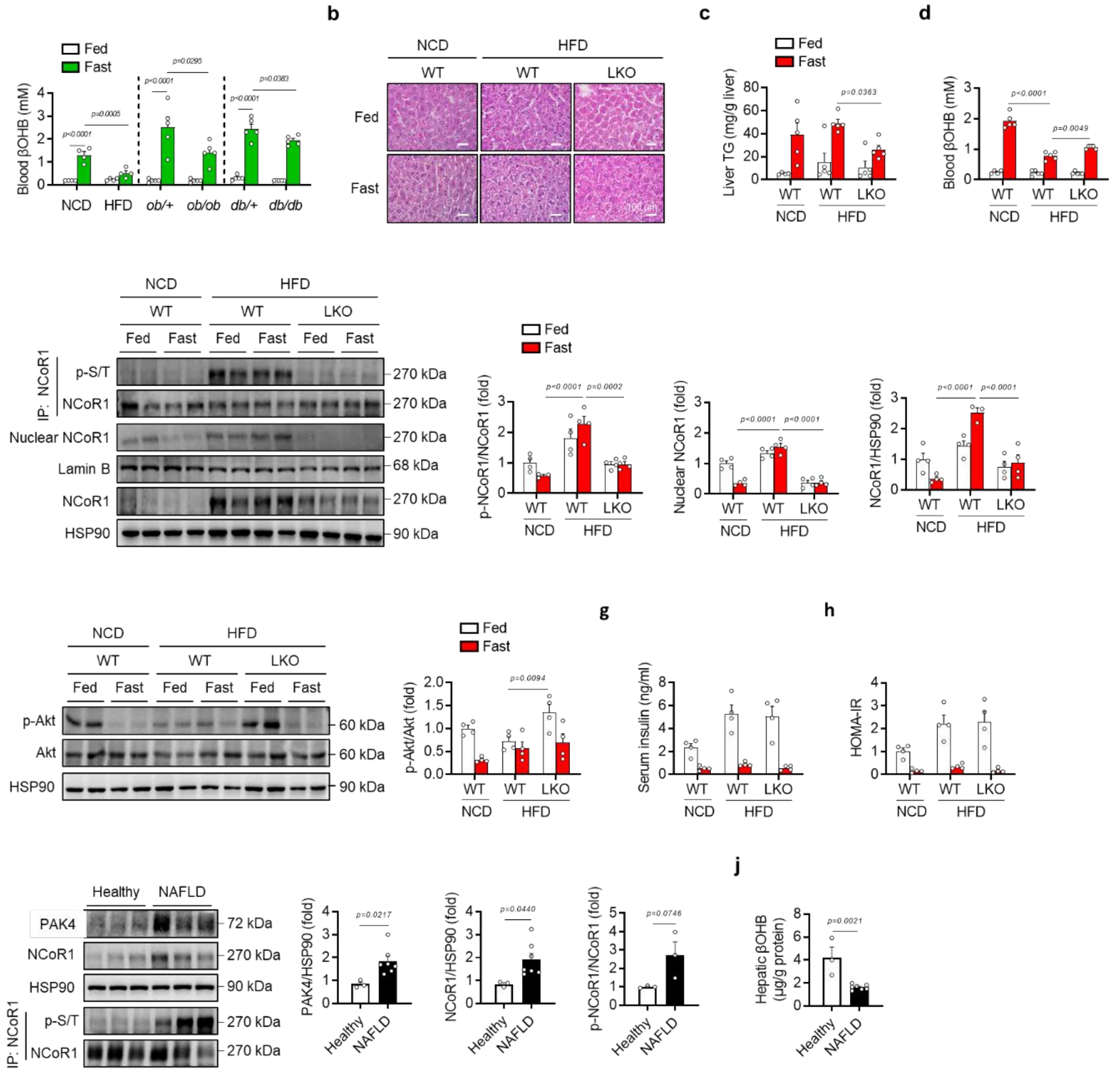

Supplementary Figure 7. Defective ketogenesis in mice with fatty liver and NAFLD patients, associated with upregulations of PAK4 and NCoR1. a. Blood levels of βOHB were analyzed in mice from 12-week HFD-fed (*n* = 4), *ob/ob* (*n* = 5), and *db/db* (*n* = 5) male mice after *ad libitum* feeding (Fed) or 24-h fasting (Fast). **b-f**. Eight-week-old male *Pak4* LKO and WT mice were fed normal chow or HFD for 5 weeks. Hematoxylin and eosin (H&E) staining of liver sections (b, scale bars, 100 μm), liver TG levels (c, *n* = 4 for NCD Fed, *n* = 5 for other groups), blood levels of βOHB (d, *n* = 4 for NCD Fed, *n* = 5 for other groups) and Western blot analysis of total- and phopho-NCoR1 (e, *n* = 4), phospho-Akt (f, *n* = 4) in liver tissues. **g**.

Serum levels of insulin were analyzed by ELISA (*n* = 4). **h**. Homeostasis Model Assessment-insulin resistance (HOMA-IR) was calculated using the following formula: HOMA-IR = fasting glucose (mg/dl) × insulin (μU/ml)/405 (*n* = 4). **i**, **j**. PAK4 (*n* = 3 for Healthy and *n* =7 for NAFLD) and total- (*n* = 3 for Healthy and *n* =7 for NAFLD) and phospho-NCoR1(*n* = 3) protein levels (i) and βOHB levels in liver tissues (j,*n* = 3 for Healthy and *n* =7 for NAFLD) from healthy adults or NAFLD patients. Data are presented as the mean ± SEM. Two-way ANOVA followed by Tukey's multiple comparisons test (a, c-h), and unpaired two-tailed *t* test (i, j) were conducted for statistical analyses. Source data are provided as a Source Data file.

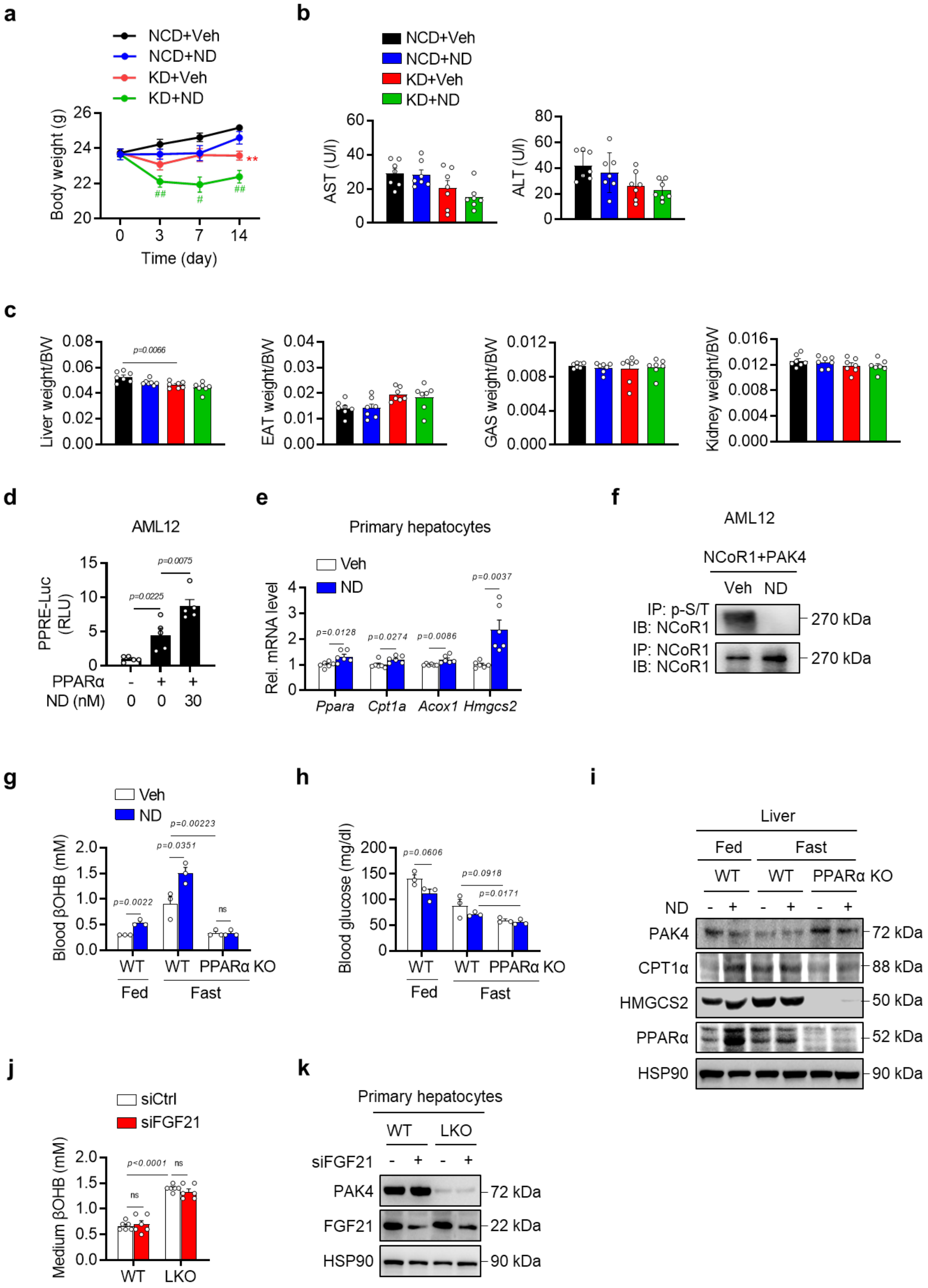
a
NCD+Veh
NCD+ND
KD+Veh
KD+ND
Body weight (g)
Time (day)
b
AST (U/l)
ALT (U/l)
c
p=0.0066
Liver weight/BW
EAT weight/BW
GAS weight/BW
Kidney weight/BW
d
AML12
PPRE-Luc (RLU)
p=0.0225
p=0.0075
PPARα
ND (nM)
e
Primary hepatocytes
Veh
ND
Rel. mRNA level
p=0.0128
p=0.0274
p=0.0086
p=0.0037
Ppara
Cpt1a
Acox1
Hmgcs2
f
AML12
NCoR1+PAK4
Veh
ND
IP: p-S/T
IB: NCoR1
IP: NCoR1
IB: NCoR1
270 kDa
g
Veh
ND
Blood βOHB (mM)
p=0.0022
p=0.0351
p=0.00223
ns
WT
PPARα KO
Fed
Fast
h
Blood glucose (mg/dl)
p=0.0606
p=0.0918
p=0.0171
i
Liver
Fed
Fast
WT
PPARα KO
ND
PAK4
CPT1α
HMGCS2
PPARα
HSP90
72 kDa
88 kDa
50 kDa
52 kDa
90 kDa
j
siCtrl
siFGF21
Medium βOHB (mM)
p<0.0001
ns
WT
LKO
k
Primary hepatocytes
WT
LKO
siFGF21
PAK4
FGF21
HSP90
72 kDa
22 kDa
90 kDa

Supplementary Figure 8. Metabolic phenotypes in male C57BL/6 mice or *Ppara* KO mice administered with PAK4 inhibitor ND201651 (compare to Figure 6). a. Body weights of mice fed a normal chow or ketogenic diet (KD) during ND201651 (ND, 50 mg/kg) treatment were monitored at the indicated time periods (*n* = 7). $^{*}p<0.05$ versus NCD+Veh; $^{\#}p<0.05$ versus KD+Veh. **b**, **c**. Serum levels of AST and ALT (b) and tissue weights of the liver, epididymal adipose tissue (EAT), gastrocnemius (GAS), and kidney normalized against body weight (c) were determined after 2 weeks of ND201651 treatment (*n* = 7). **d**. AML12 cells were co-transfected with PPARα expression plasmid and PPRE promoter luciferase plasmid, and subsequently treated with ND201651 (30 nM) for 24 h. PPRE-luciferase activities were measured and expressed as the fold change relative to mock (*n* = 5). **e**. Mouse primary hepatocytes were treated with ND201651 (30 nM) for 24 h, and mRNA levels of β-oxidation and ketogenesis-related genes were analyzed by qPCR (*n* = 6). **f**. AML12 cells were treated with ND201651 (30 nM) for 24 h after co-transfection with PAK4 and NCoR1. Phosphorylation of NCoR1 was analyzed by immunoblotting of NCoR1 following immunoprecipitation with anti- p-Ser/Thr antibody. **g-i**. *Ppara* KO mice and their littermates (WT) were orally administered ND201651 (50 mg/kg) once a day for 3 days. Blood levels of βOHB (g, *n* = 3) and glucose (h, *n* = 3), and protein levels of CPT1α, HMGCS2 and PPARα in liver tissues were analyzed after *ad libitum* feeding or 24 h fasting (i). **j**, **k**. *Pak4* LKO and WT hepatocytes were transfected with siRNA targeting FGF21 or control siRNA, and the medium βOHB concentration was assessed (j, *n* = 6). Successful depletion of FGF21 was validated by Western blotting (k). Data are presented as the mean ± SEM. One-way ANOVA followed by Tukey's multiple comparisons test (a–d) and unpaired two-tailed *t* test (e, g, h, j) were conducted for statistical analyses. Source data are provided as a Source Data file.

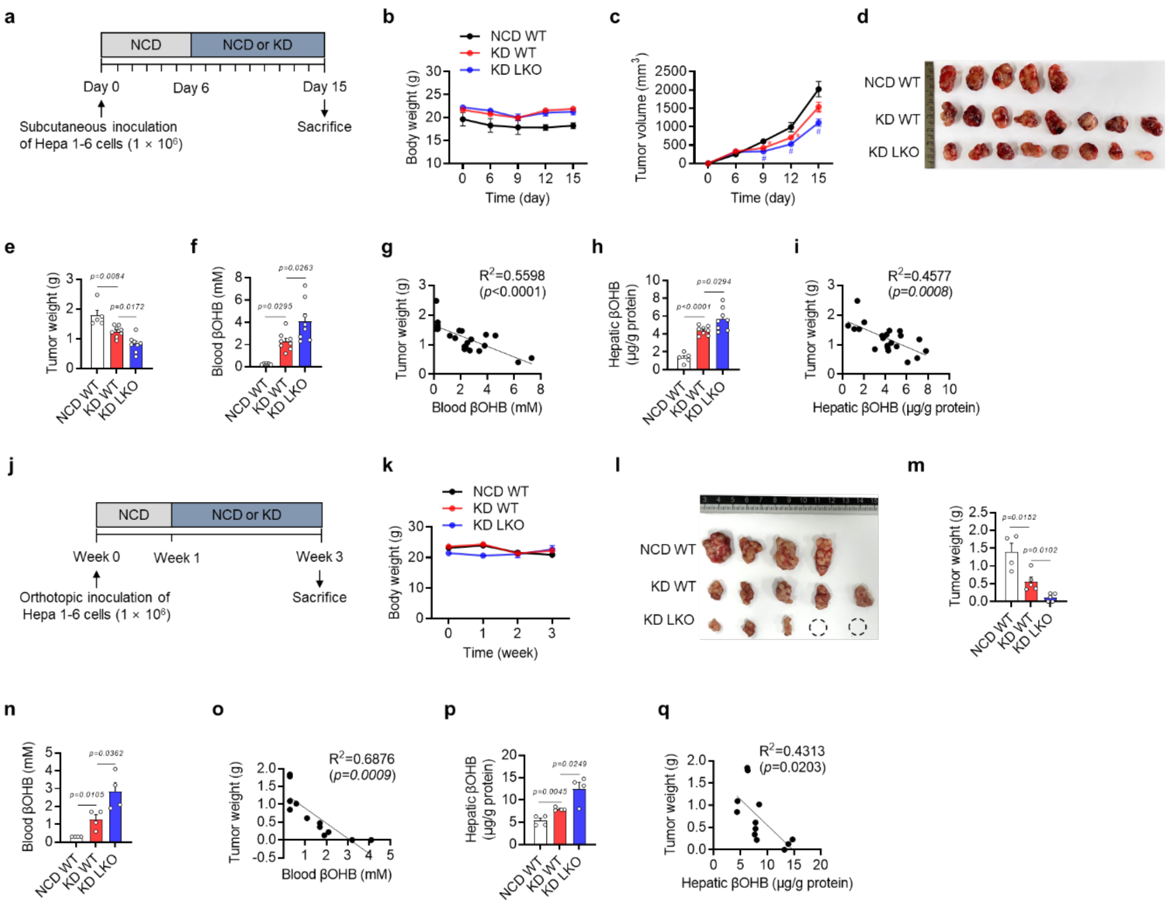

Supplementary Figure 9. Suppression of extra- and intra-hepatic tumor growth in *Pak4* LKO mice. a, **j**. Schematic of the extra- (a) and intra-hepatic (j) tumor implantation models using male *Pak4* LKO and WT mice fed a normal chow diet (NCD) or ketogenic diet (KD). **b**, **c**. Body weight (b) and tumor growth (c, n = 10 for NCD and n = 18 for KD) in *Pak4* LKO and WT mice. Tumor size was measured with electronic calipers and volumes were determined using the following formula: length (mm) × width (mm)^2 × 0.5. $^*p<0.05$ versus NCD WT; $^\#p<0.05$ versus KD WT. **d**, **e**. Representative gross images of tumors (d) and tumor weights (e) at the end of the study. **f**, **g**. Blood βOHB levels (f) and its correlation with tumor weight (g, n = 21). **h**, **i**. Hepatic βOHB levels (h) and its correlation with tumor weight (i, n = 21). n = 5 for NCD and n = 8 for KD in b, e, f and h. **k-m**. Body weight change (k), gross images of tumors (l) and tumor weights (m) at the end of the study. n = 4 for NCD and n =5 for KD in k and m. **n**, **o**. blood βOHB levels (n, n = 4) and its correlation with tumor weight (o, n = 12). **p**, **q**.

Hepatic βOHB levels (p, n = 4) and its correlation with tumor weight (q, n = 12). Data are presented as the mean ± SEM. One-way ANOVA followed by Tukey's multiple comparisons test (b, c, e, f, h, k, m, n, p) was conducted for statistical analyses. The Pearson correlation coefficients were calculated between continuous variables (g, i, o, q). Source data are provided as a Source Data file.

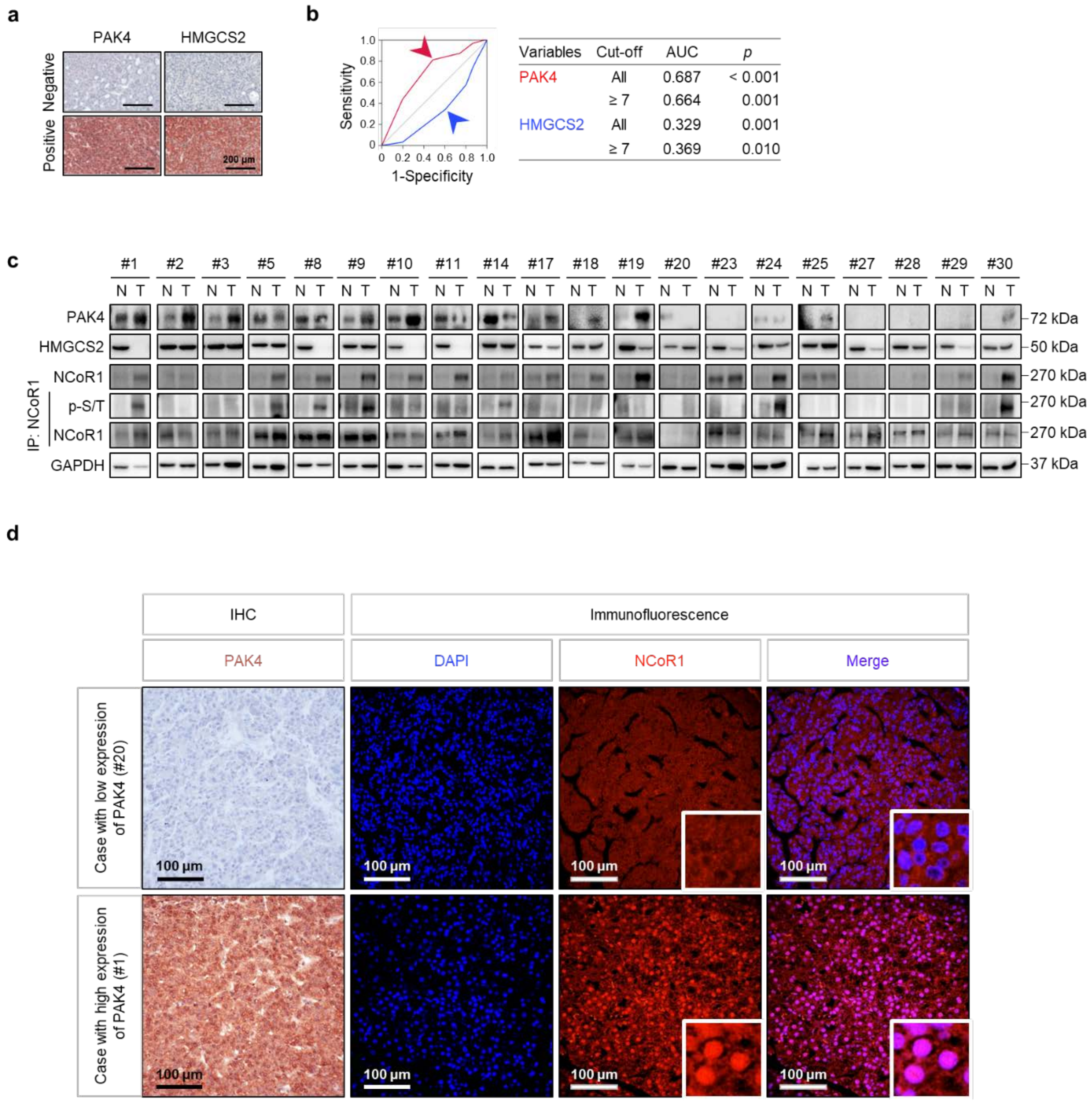

Supplementary Figure 10 (compare to Figure 7). Hepatic expression of PAK4, HMGCS2, and NCoR1 in hepatocellular carcinoma (HCC) patients. a. Representative immunohistochemical staining images for PAK4 and HMGCS2 in liver sections of patients with HCC. **b.** The cut-off points for PAK4 and HMGCS2 expression were determined based on a receiver operating characteristic curve analysis. The cut-off points for PAK4 (red arrow) and HMGCS2 (blue arrow) were both 7 (AUC, area under the curve). **c**. Immunoblotting for the indicated proteins in non-tumor (N) and tumor (T) liver tissues in HCC patients. NCoR1

phosphorylation was analyzed by immunoblotting with anti-p-Ser/Thr antibody following immunoprecipitation with anti-NCoR1 antibody. GAPDH was used as a loading control. **d**. The expression of NCoR1 in the nucleus in cases of low- and high-expression of PAK4 in HCC tissues. The *p*-value in ROC curve (b) analysis is one-sided.

2. Supplementary Tables

Supplementary Table 1. Clinicopathologic variables and the expression of PAK4 and HMGCS2 in 130 hepatocellular carcinomas

Characteristics		No.	PAK4		HMGCS2	
			Positive	*p*	Positive	*p*
Sex	Male	114	77 (68%)	0.062	52 (46%)	0.205
	Female	16	7 (44%)		10 (63%)	
Age (year)	≤ 60	76	44 (58%)	0.057	40 (53%)	0.181
	> 60	54	40 (74%)		22 (41%)	
AFP (ng/ml)	< 100	92	58 (63%)	0.560	44 (48%)	0.962
	≥ 100	38	26 (68%)		18 (47%)	
HBV	Negative	36	23 (64%)	0.915	13 (36%)	0.102
	Positive	94	61 (65%)		49 (52%)	
HCV	Negative	122	79 (65%)	0.897	59 (48%)	0.551
	Positive	8	5 (63%)		3 (38%)	
Liver cirrhosis	Absence	70	51 (73%)	0.034	34 (49%)	0.828
	Presence	60	33 (55%)		28 (47%)	
Bilirubin (mg/dl)	< 0.7	59	38 (64%)	0.964	28 (47%)	0.961
	≥ 0.7	71	46 (65%)		34 (48%)	
Albumin (mg/dl)	< 3.5	116	77 (66%)	0.226	57 (49%)	0.342
	≥ 3.5	14	7 (50%)		5 (36%)	
Tumor stage	I	50	27 (54%)	0.023	22 (44%)	0.220
	II	49	31 (63%)		28 (57%)	
	III & IV	31	26 (84%)		12 (39%)	
Histologic grade	Low	78	49 (63%)	0.600	37 (47%)	0.943
	High	52	35 (67%)		25 (48%)	
HMGCS2	Negative	68	43 (63%)	0.730		
	Positive	62	41 (66%)			

Abbreviations: AFP, α-fetoprotein; HBV, hepatitis B virus; HCV, hepatitis C virus

The p-value in ROC curve analysis is two-sided

Supplementary Table 2. Univariate and multivariate Cox regression analysis for the survival in hepatocellular carcinoma patients

Characteristics	No.	OS		RFS	
		HR (95% CI)	*p*	HR (95% CI)	*p*
Univariate Cox regression analysis					
Sex, male (vs female)	114/130	0.691 (0.298-1.604)	0.390	0.880 (0.441-1.758)	0.717
Age, > 60 (vs ≤ 60)	54/130	1.823 (1.109-2.995)	0.018	1.580 (1.032-2.417)	0.035
AFP, ≥ 100 ng/ml (vs < 100 ng/ml)	38/130	1.727 (1.035-2.880)	0.036	1.988 (1.283-3.081)	0.002
Albumin, < 3.5 mg/dl (vs ≥ 3.5 mg/dl)	116/130	1.812 (0.892-3.681)	0.100	1.582 (0.839-2.982)	0.156
Bilirubin, ≥ 0.7 mg/dl (vs < 0.7 mg/dl)	71/130	1.102 (0.673-1.804)	0.699	1.019 (0.668-1.554)	0.931
Tumor stage, I	50/130	1	0.002	1	< 0.001
II	49/130	1.713 (0.919-3.193)	0.090	1.633 (0.973-2.743)	0.064
III & IV	31/130	3.109 (1.658-5.830)	< 0.001	3.068 (1.782-5.283)	< 0.001
Histologic grade, high (vs low)	52/130	1.429 (0.869-2.350)	0.160	1.508 (0.983-2.312)	0.060
HMGCS2, negative (vs positive)	68/130	1.877 (1.118-3.149)	0.017	1.540 (1.001-2.369)	0.049
PAK4, positive (vs negative)	84/130	3.082 (1.643-5.781)	< 0.001	2.770 (1.661-4.618)	< 0.001
Multivariate Cox regression analysis*					
Age, > 60 (vs ≤ 60)				1.780 (1.110-2.854)	0.017
AFP, ≥ 100 ng/ml (*vs* < 100 ng/ml)				1.981 (1.245-3.151)	0.004
Tumor stage, I		1	0.051	1	0.013
II		1.630 (0.870-3.052)	0.127	1.359 (0.801-2.307)	0.256
III & IV		2.237 (1.169-4.281)	0.015	2.337 (1.314-4.157)	0.004
HMGCS2, negative (vs positive)		1.989 (1.180-3.351)	0.010		
PAK4, positive (vs negative)		2.710 (1.414-5.191)	0.003	2.235 (1.317-3.793)	0.003

Abbreviations: HBV, hepatitis B virus; HCV, hepatitis C virus; AFP, α-fetoprotein; HR, hazard ratio; 95% CI, 95% confidence interval. * Variables considered analysis were age, TNM stage, AFP level, and the expression of PAK4 and HMGCS2.

In multivariate survival analysis with Cox regression analysis, adjustments are made for multiple comparisons by using Bonferroni correction. Multivariate analysis was performed with the factors significantly associated with OS and RFS in univariate analysis.

Supplementary Table 3. Antibodies used for Western blotting, immunofluorescence and immunohistochemical analyses

Antibody	Catalogue No.	Dilution Fold	Company
Western Blot			
PAK4 (G222)	62690	1:2500	Cell Signaling Technology
p-PAK4 (S474)	3241	1:2500	Cell Signaling Technology
HSP90 (C45G5)	4877	1:2500	Cell Signaling Technology
Ubiquitin (E4I2J)	43124	1:2500	Cell Signaling Technology
HMGCS2 (D3U1A)	20940	1:2500	Cell Signaling Technology
Phospho-PKA Substrate (100G7E)	9624	1:2500	Cell Signaling Technology
Phospho-(Ser/Thr)	9631	1:2500	Cell Signaling Technology
Sirt1	2310	1:2500	Cell Signaling Technology
Sirt6 (D8D12)	12486	1:2500	Cell Signaling Technology
Phospho-Akt (Ser473) (D9E)	4060	1:2500	Cell Signaling Technology
Akt	9272	1:2500	Cell Signaling Technology
Acetylated-Lysine	9441	1:2500	Cell Signaling Technology
CHOP (L63F7)	2895	1:2500	Cell Signaling Technology
Phospho-S6 Ribosomal Protein (Ser240/244)	2215	1:2500	Cell Signaling Technology
S6 Ribosomal Protein (5G10)	2217	1:2500	Cell Signaling Technology
NCoR1	5948	1:2500	Cell Signaling Technology
NEDD4	2740	1:2500	Cell Signaling Technology
ATF-6 (D4Z8V)	65880	1:2500	Cell Signaling Technology
Phospho-PERK (Thr980) (16F8)	3179	1:2500	Cell Signaling Technology
PAK4 (B-3)	SC-390507	1:2500	Santa Cruz Biotechnology
FGF21 (H-105)	SC-292879	1:2500	Santa Cruz Biotechnology
PPARα (H-2)	SC-398394	1:2500	Santa Cruz Biotechnology
Sirt4	3224	1:2500	Bioworld Technology
LaminB1 (L75)	BS3547	1:2500	Bioworld Technology
GAPDH (A531)	AP0066	1:2500	Bioworld Technology
Sirt5 (aa30-46)	LS-B2060	1:2500	LifeSpan Biosciences
Sirt7 (aa317-366)	LS-B1566	1:2500	LifeSpan Biosciences
FoxO1 (Acetyl-Lys294)	LS-C800723	1:2500	LifeSpan Biosciences
Sirt2 (EPR20411-105)	ab211033	1:2500	Abcam
Sirt3	ab189860	1:2500	Abcam
GRP78 BiP	ab21685	1:2500	Abcam
NCOR2/SMRT	ab24551	1:2500	Abcam
CPT1A (8F6AE9)	ab128568	1:2500	Abcam
T-OXPHOS	ab110413	1:2500	Abcam
THRβ	ab53170	1:2500	Abcam
p-IRE1α	ab48187	1:2500	Abcam
MDM2 (2A10)	ab16895	1:2500	Abcam
LXRα	14351-1-AP	1:2500	Proteintech
Proximity Ligation Assay			
NCoR1	5948	1:100	Cell Signaling Technology
p300 (D8Z4E)	86377	1:100	Cell Signaling Technology
PPARα (H-2)	SC-398394	1:100	Santa Cruz Biotechnology
Immunofluorescence			
NCoR1	5948	1:100	Cell Signaling Technology
PPARα (H-2)	SC-398394	1:100	Santa Cruz Biotechnology

Supplementary Table 4. Information for primers (forward, FOR; reverse, REV)

Gene (qPCR)	Sequences for primers	Accession No.
Pak4	FOR: GCTCCCCTTTGAAGATGTCA<br>REV: GACCCACAAGGACTCAAGGA	NM_027470
Ppara	FOR: CAGTGGGGAGAGAGGACAGA<br>REV: AGTTCGGGAACAAGACGT TG	NM_133947
Cpt1a	FOR: TTGGAAGTCTCCCTCCTTCA<br>REV: GCCCATGTTGTACAGCTTCC	NM_013495
Acox1	FOR: CCTGATTCAGCAAGGTAGGG<br>REV: TCGCAGACCCTGAAGAAATC	NM_015729
Hmgcs2	FOR: TGGTTCAAGACAGGGACACAGAAC<br>REV: AGAGGAATACCAGGGCCCAACAAT	NM_008256
Acat1	FOR: GGCTGGTGCAGGAAATAAGA<br>REV: GGAATCCCTGCCTTTTCAAT	NM_144784
Hmgcl	FOR: ACCACCAGCTTTGTGTCTCC<br>REV: GAGGCAGCTCCAAAGATGAC	NM_008254
Bdh1	FOR: GCTTCCAGCGTCAAAGGAGTT<br>REV: CAGTTGCGAATCTTCCCGTC	NM_175177
Acaca	FOR: ACCGCAAGCAGATCTACAAC<br>REV: TGGGGTGCAGCTTGTACA	NM_133360
Fasn	FOR: GGAGGTGGTGATAGCCGGTAT<br>REV: TGGGTAATCCATAGAGCCCAG	NM_007988
Nr1h3	FOR: AAGCCCTGCATGCCTACGT<br>REV: TGCAGACGCAGTGCAAACA	NM_001177730
Scd1	FOR: CCATCGCCTGTGGAGTCAC<br>REV: GTCGGATAAATCTAGCGTAGCA	NM_009127
Srebf1	FOR: ACAGCCGCCCTTCAAGTG<br>REV: TCACAGGCATTGTGGTCAGAA	NM_001313979
Tnf	FOR: CCCGAGTGACAAGCCTGTAG<br>REV:GATGGCAGAGAGGAGGTTGAC	NM_001278601
Ccl2	FOR: AAAGTCTCTGCCGCCCTTCT<br>REV:GATTGCATCTGGCTGAGCG	NM_011333
Il6	FOR: ACAGCCACTCACCTCTTCAG<br>REV: CCATCTTTTTCAGCCATCTTT	NM_001314054
Il1b	FOR: AGATGATAAGCCCACTCTACAG<br>REV:ACATTCAGCACAGGACTCTC	NM_008361
Ccl5	FOR: GATGGACATAGAGGACACAACT<br>REV:TGGGACGGCAGATCTGAGGG	NM_013653
Actb	FOR: ATGGAGGGGAATACAGCC<br>REV: TTCTTTGCAGCTCCTTCGTT	NM_007393
Gene (Chip)	Sequences for primers	
Ppara PPRE	FOR: TTCCGAACCATTCTTTCCAG<br>REV: GCTGCCTTCTTTTGCAGAGT	
Cpt1a PPRE	FOR: CTTTCCTACTGAGGCCCAGATAG<br>REV: TACAGCCTAGAACCCTGACTG	
Hmgcs2 PPRE	FOR: TGAGCCACTCAGCAGAGGAATCAG<br>REV: CTGGGTTGGGCTTTATAAGACTCC	

Supplementary Table 5. Identification of phosphorylation sites in NCoR1 by LC-MS/MS

Sequence	Charge state	Monoisotopic mass
QT(p)ILNDYITSQQMQVNLR	$[M+2H]^{2+}$	1123.0378
SPESQAQT(p)VLHPRPGSR	$[M+3H]^{3+}$	642.9776
