# Supplementary material for: p21-activated kinase 4 suppresses fatty acid β-oxidation and ketogenesis by phosphorylating NCoR1
Source: Nat Commun. 2023 Aug 17;14:4987. doi: 10.1038/s41467-023-40597-z (PMC10435519; doi:10.1038/s41467-023-40597-z)
Supplement: Supplementary file 3 — Reporting Summary [file 41467_2023_40597_MOESM3_ESM.pdf]

nature portfolio

Corresponding author(s): Eun Ju Bae
Byung-Hyun Park

Last updated by author(s): Jul 25, 2023

Reporting Summary

Nature Portfolio wishes to improve the reproducibility of the work that we publish. This form provides structure for consistency and transparency in reporting. For further information on Nature Portfolio policies, see our Editorial Policies and the Editorial Policy Checklist.

Statistics

For all statistical analyses, confirm that the following items are present in the figure legend, table legend, main text, or Methods section.

n/a	Confirmed	
[ ]	[x]	The exact sample size (*n*) for each experimental group/condition, given as a discrete number and unit of measurement
[ ]	[x]	A statement on whether measurements were taken from distinct samples or whether the same sample was measured repeatedly
[ ]	[x]	The statistical test(s) used AND whether they are one- or two-sided<br>*Only common tests should be described solely by name; describe more complex techniques in the Methods section.*
[ ]	[ ]	A description of all covariates tested
[ ]	[x]	A description of any assumptions or corrections, such as tests of normality and adjustment for multiple comparisons
[ ]	[x]	A full description of the statistical parameters including central tendency (e.g. means) or other basic estimates (e.g. regression coefficient) AND variation (e.g. standard deviation) or associated estimates of uncertainty (e.g. confidence intervals)
[ ]	[x]	For null hypothesis testing, the test statistic (e.g. *F*, *t*, *r*) with confidence intervals, effect sizes, degrees of freedom and *P* value noted<br>*Give P values as exact values whenever suitable.*
[x]	[ ]	For Bayesian analysis, information on the choice of priors and Markov chain Monte Carlo settings
[x]	[ ]	For hierarchical and complex designs, identification of the appropriate level for tests and full reporting of outcomes
[ ]	[x]	Estimates of effect sizes (e.g. Cohen's *d*, Pearson's *r*), indicating how they were calculated

Our web collection on statistics for biologists contains articles on many of the points above.

Software and code

Policy information about availability of computer code

Data collection	RNA Seq was done by the Illumina Hiseq 4000 system. Microscopic imaging was done by Leica DM750 microscope.
Data analysis	RNA-seq: STAR RNA-weq aligner sorfware (version STAR_2.5.0a)<br>Gene Set Enrichment Analysis (GSEA, Broad Institute, version 4.1.0)<br>Gene interaction network (Cytoscape, version 3.8.2)<br>Western blot analysis: Las-4000 imager (version 1.2)<br>Plotting and Statistics: SPSS software (version 20.0) and Graphpad Prism (version 9.4)

For manuscripts utilizing custom algorithms or software that are central to the research but not yet described in published literature, software must be made available to editors and reviewers. We strongly encourage code deposition in a community repository (e.g. GitHub). See the Nature Portfolio guidelines for submitting code & software for further information.

Data

Policy information about availability of data

All manuscripts must include a data availability statement. This statement should provide the following information, where applicable:

- Accession codes, unique identifiers, or web links for publicly available datasets
- A description of any restrictions on data availability
- For clinical datasets or third party data, please ensure that the statement adheres to our policy

The source data for all box plots, bars, and line graphs as well as the original uncropped Western blots can be found in the online Source Data File. Raw and processed RNA-seq datasets were deposited into the NCBI GEO database under the accession number GSE214442.

Research involving human participants, their data, or biological material

Policy information about studies with human participants or human data. See also policy information about sex, gender (identity/presentation), and sexual orientation and race, ethnicity and racism.

Reporting on sex and gender	We did not consider sex in human tissue analysis.
Reporting on race, ethnicity, or other socially relevant groupings	We did not consider race, ethnicity and other socially relevant factors.
Population characteristics	Blood and liver tissues are obtained from healthy volunteer, NAFLD patients or hepatocellular carcinoma patients.
Recruitment	The biospecimens and data used in this study were provided by the Biobank of Jeonbuk National University Hospital. participants were selected randomly and without self-selection.
Ethics oversight	Jeonbuk National University Hospital (IRB number CUH 2021-05-029, approved on 18 June 2021)

Note that full information on the approval of the study protocol must also be provided in the manuscript.

Field-specific reporting

Please select the one below that is the best fit for your research. If you are not sure, read the appropriate sections before making your selection.

☒ Life sciences ☐ Behavioural & social sciences ☐ Ecological, evolutionary & environmental sciences

For a reference copy of the document with all sections, see nature.com/documents/nr-reporting-summary-flat.pdf

Life sciences study design

All studies must disclose on these points even when the disclosure is negative.

Sample size	For in vitro and in vivo experiments, sample size selection was based on literature (PMID: 30530497, 21179166, 22183976, 25998209) and the lab's previous experience. We usually observed standard deviation of around 20% and therefore average of 6 mice per group is sufficient to detect 33% change with 80% power. For signaling assays, we typically performed at least three independent experiment repeats to allow statistical analysis and robust conclusions to be drawn. For RNA-seq analysis, the sample size was determined by minimum requirement for statistical analysis (three samples).
Data exclusions	No data exclusions in this manuscript.
Replication	For all experiments we used multiple biological replicates, as indicated in the figure legends. For quantitative measurements, three or more independent experiments were carried out and statistical analysis performed. For non-quantitative experiments, each experimental condition was repeated at least one more time to confirm the observation.
Randomization	Investigators were blinded to group allocation during data collection and data analyses.
Blinding	*Describe whether the investigators were blinded to group allocation during data collection and/or analysis. If blinding was not possible, describe why OR explain why blinding was not relevant to your study.*

Reporting for specific materials, systems and methods

We require information from authors about some types of materials, experimental systems and methods used in many studies. Here, indicate whether each material, system or method listed is relevant to your study. If you are not sure if a list item applies to your research, read the appropriate section before selecting a response.

Materials & experimental systems

n/a	Involved in the study
☐	☒ Antibodies
☐	☒ Eukaryotic cell lines
☒	☐ Palaeontology and archaeology
☐	☒ Animals and other organisms
☒	☐ Clinical data
☒	☐ Dual use research of concern
☒	☐ Plants

Methods

n/a	Involved in the study
☒	☐ ChIP-seq
☒	☐ Flow cytometry
☒	☐ MRI-based neuroimaging

Antibodies

Antibodies used

All antibodies used for Western blotting, immunofluorescence, and chromatin immunoprecipitation analyses are provided as follows:

Western blot
PAK4 (G222) (62690, 1:2500 dilution, Cell Signaling Technology)
Phospho-PAK4 (Ser474) (3241, 1:2500 dilution, Cell Signaling Technology)
HSP90 (C45G5) (4877,1:2500 dilution, Cell Signaling Technology)
Ubiquitin (E4I2J) (43124,1:2500 dilution, Cell Signaling Technology)
HMGCS2 (D3U1A) (20940, 1:2500 dilution, Cell Signaling Technology)
Phospho-PKA Substrate (100G7E) (9624, 1:2500 dilution, Cell Signaling Technology)
Phospho-(Ser/Thr) (9631, 1:2500 dilution, Cell Signaling Technology)
Sirt1 (2310, 1:2500 dilution, Cell Signaling Technology)
Sirt6 (D8D12) (12486, 1:2500 dilution, Cell Signaling Technology)
Phospho-Akt (Ser473) (D9E) (4060, 1:2500 dilution, Cell Signaling Technology)
Akt (9272, 1:2500 dilution, Cell Signaling Technology)
Acetylated-Lysine (9441, 1:2500 dilution, Cell Signaling Technology)
CHOP (L63F7) (2895, 1:2500 dilution, Cell Signaling Technology)
Phospho-S6 Ribosomal Protein (Ser240/244) (2215, 1:2500 dilution, Cell Signaling Technology)
S6 Ribosomal Protein (5G10) (2217, 1:2500 dilution, Cell Signaling Technology)
NCoR1 (5948, 1:2500 dilution, Cell Signaling Technology)
NEDD4 (2740, 1:2500 dilution, Cell Signaling Technology)
ATF-6 (D4Z8V) (65880, 1:2500 dilution, Cell Signaling Technology)
Phospho-PERK (Thr980) (16F8) (3179, 1:2500 dilution, Cell Signaling Technology)
PAK4 (B-3) (SC-390507, 1:2500 dilution, Santa Cruz Biotechnology)
FGF21 (H-105) (SC-292879, 1:2500 dilution, Santa Cruz Biotechnology)
PPARα (H-2) (SC-398394, 1:2500 dilution, Santa Cruz Biotechnology)
Sirt4 (3224, 1:2500 dilution, Bioworld Technology)
Lamin B1 (L75) (BS3547, 1:2500 dilution, Bioworld Technology)
GAPDH (A531) (AP0066, 1:2500 dilution, Bioworld Technology)
Sirt5 (aa30-46) (LS-B2060, 1:2500 dilution, LifeSpan Biosciences)
Sirt7 (aa317-366) (LS-B1566, 1:2500 dilution, LifeSpan Biosciences)
FoxO1 (Acetyl-Lys294) (LS-C800723, 1:2500 dilution, LifeSpan Biosciences)
Sirt2 (EPR20411-105) (ab211033, 1:2500 dilution, Abcam)
Sirt3 (ab189860, 1:2500 dilution, Abcam)
GRP78 BiP (ab21685, 1:2500 dilution, Abcam)
NCOR2/SMRT (ab24551, 1:2500 dilution, Abcam)
CPT1A (8F6AE9) (ab128568, 1:2500 dilution, Abcam)
T-OXPHOS (ab110413, 1:2500 dilution, Abcam)
THRβ (ab53170, 1:2500 dilution, Abcam)
p-IRE1α (ab48187, 1:2500 dilution, Abcam)
MDM2 (2A10) (ab16895, 1:2500 dilution, Abcam)
LXRα (14351-1-AP, 1:2500 dilution, Proteintech)
Proximity Ligation Assay
NCoR1 (5948, 1:100 dilution, Cell Signaling Technology)
p300 (D8Z4E) (86377, 1:100 dilution, Cell Signaling Technology)
PPARα (H-2) (SC-398394, 1:100 dilution, Santa Cruz Biotechnology)

Immunofluorescence
NCoR1 (5948, 1:100 dilution, Cell Signaling Technology)
PPARα(H-2) (SC-398394, 1:100 dilution, Santa Cruz Biotechnology)
Alexa Fluor 488-conjugated goat anti-mouse IgG1 (11001, 1:100 dilution, Thermo Fisher Scientific)
Alexa Fluor 594-conjugated goat anti-rabbit IgM (11012, 1:100 dilution, Thermo Fisher Scientific)

Validation	All antibodies used in this study were commercially developed and validated by the companies.<br>PAK4 (G222), https://www.cellsignal.com/products/primary-antibodies/pak4-g222-antibody/62690<br>Phospho-PAK4 (Ser474), https://www.cellsignal.com/products/primary-antibodies/phospho-pak4-ser474-pak5-ser602-pak6-ser560-antibody/3241<br>HSP90(C45G5), https://www.cellsignal.com/products/primary-antibodies/hsp90-c45g5-rabbit-mab/4877<br>Ubiquitin(E4I2J), https://www.cellsignal.com/products/primary-antibodies/ubiquitin-e4i2j-rabbit-mab/43124?site-search-type=Products&N=4294956287&Ntt=ubiquitin&fromPage=plp<br>HMGCS2(D3U1A), https://www.cellsignal.com/products/primary-antibodies/hmgcs2-d3u1a-rabbit-mab/20940?site-search-type=Products&N=4294956287&Ntt=hmgcs2%28d3u1a%29&fromPage=plp&_requestid=464612<br>Phospho-PKA Substrate(100G7E), https://www.cellsignal.com/products/primary-antibodies/phospho-pka-substrate-rrxs-t-100g7e-rabbit-mab/9624?_=1664199480543&Ntt=p-PKA%20substrate&tahead=true<br>Phospho-(Ser/Thr) Phe, https://www.cellsignal.com/product/productDetail.jsp?productId=9631<br>Sirt1, https://www.cellsignal.com/product/productDetail.jsp?productId=2310<br>Sirt6(D8D12), https://www.cellsignal.com/product/productDetail.jsp?productId=12486<br>Phospho-Akt (Ser473) (D9E), https://www.cellsignal.com/product/productDetail.jsp?productId=4060<br>Akt, https://www.cellsignal.com/product/productDetail.jsp?productId=9272<br>Acetylated-Lysine, https://www.cellsignal.com/products/primary-antibodies/acetylated-lysine-antibody/9441?site-search-type=Products&N=4294956287&Ntt=acetyl+lysine&fromPage=plp<br>CHOP(L63F7),https://www.cellsignal.com/products/primary-antibodies/chop-l63f7-mouse-mab/2895?site-search-type=Products&N=4294956287&Ntt=chop&fromPage=plp<br>Phospho-S6 Ribosomal Protein (Ser240/244), https://www.cellsignal.com/product/productDetail.jsp?productId=2215<br>S6 Ribosomal Protein (5G10), https://www.cellsignal.com/product/productDetail.jsp?productId=2217<br>NCoR1, https://www.cellsignal.com/products/primary-antibodies/ncor1-antibody/5948?site-search-type=Products&N=4294956287&Ntt=ncor1&fromPage=plp<br>NEDD4,https://www.cellsignal.com/products/primary-antibodies/nedd4-antibody/2740<br>ATF-6 (D4Z8V), https://www.cellsignal.com/products/primary-antibodies/atf-6-d4z8v-rabbit-mab/65880?site-search-type=Products&N=4294956287&Ntt=atf6&fromPage=plp<br>Phospho-PERK (Thr980) (16F8), https://www.cellsignal.com/products/primary-antibodies/phospho-perk-thr980-16f8-rabbit-mab/3179?site-search-type=Products&N=4294956287&Ntt=p-perk&fromPage=plp<br>PAK4(B-3),https://www.scbt.com/p/pak4-antibody-b-3<br>FGF21(H-105),chttps://www.citeab.com/antibodies/793317-sc-292879-fgf-21-antibody-h-10https://www.citeab.com/antibodies/793317-sc-292879-fgf-21-antibody-h-105<br>PPARα(H-2), https://www.scbt.com/ko/p/pparalpha-antibody-h-2<br>Sirt4, https://www.biovision.com/sirt4-antibody.html<br>Lamin B1 (L75), https://www.antibodypedia.com/gene/3937/LMNB1/antibody/1562122/BS3547<br>GAPDH(A531), https://www.citeab.com/antibodies/2207025-ap0066-gapdh-a531-polyclonal-antibody-n-a<br>Sirt5, https://www.lsbio.com/antibodies/ihc-plus-sirt5-antibody-sirtuin-5-antibody-aa30-46-ihc-wb-western-ls-b2060/53010<br>Sirt7, https://www.lsbio.com/antibodies/ihc-plus-sirt7-antibody-sirtuin-7-antibody-aa317-366-ihc-wb-western-ls-b1566/45501<br>FoxO1(Acetyl-Lys294), https://www.lsbio.com/antibodies/foxo1-antibody-fkhr-antibody-acetyl-lys294-ihc-wb-western-ls-c800723/827288<br>Sirt2 (EPR20411-105), https://www.abcam.com/sirt2-antibody-epr20411-105-ab211033.html<br>Sirt3, https://www.abcam.com/sirt3-antibody-ab189860.html<br>GRP78 BiP,https://www.abcam.com/products/primary-antibodies/grp78-bip-antibody-ab21685.html<br>NCOR2/SMRT, https://www.abcam.com/ncor2smrt-antibody-ab24551.html<br>CPT1A(8F6AE9), https://www.abcam.com/cpt1a-antibody-8f6ae9-ab128568.html<br>T-OXPHOS, https://www.abcam.com/products/panels/total-oxphos-rodent-wb-antibody-cocktail-ab110413.html<br>THRβ, https://www.abcam.com/products/primary-antibodies/thyroid-hormone-receptor-beta-antibody-ab53170.html<br>p-IRE1α, https://www.abcam.com/products/primary-antibodies/ire1-phospho-s724-antibody-ab48187.html<br>MDM2(2A10),https://www.abcam.com/mdm2-antibody-2a10-ab16895.html<br>LXRα , https://www.ptglab.com/products/NR1H3-Antibody-14351-1-AP.htm<br>P300, https://www.cellsignal.com/product/productDetail.jsp?productId=86377<br>Alexa Fluor 488-conjugated goat anti-mouse IgG1, https://www.thermofisher.com/antibody/product/Goat-anti-Mouse-IgG1-Cross-Adsorbed-Secondary-Antibody-Polyclonal/A-21121<br>Alexa Fluor 594-conjugated goat anti-mouse IgM, https://www.thermofisher.com/antibody/product/Goat-anti-Mouse-IgM-Heavy-chain-Cross-Adsorbed-Secondary-Antibody-Polyclonal/A-21044

Eukaryotic cell lines

Policy information about cell lines and Sex and Gender in Research

Cell line source(s)	AML12 cells (CRL-2254), HEK293T cells (CRL-3216) and Hepa 1-6 cells (CRL-1830) were purchased from ATCC.
Authentication	Cell lines were authenticated by morphology. The isolation and authentication of primary hepatocytes have been reported in our previous manuscript (10.1002/hep.32384).
Mycoplasma contamination	All cells tested negative for mycoplasma.
Commonly misidentified lines (See ICLAC register)	No commonly misidentified cell lines were used in the study.

Animals and other research organisms

Policy information about studies involving animals; ARRIVE guidelines recommended for reporting animal research, and Sex and Gender in Research

Laboratory animals	8 week-old C57BL/6 male mice were purchased from Damul Science (Daejeon, Korea).<br>8 week-old ob/+, ob/ob, db/+, and db/db male mice were obtained from Jackson Laboratory (Bar Harbor, ME, USA).<br>Pak4 LKO mice were generated by mating PAK4Pak4flox/flox mice (B6.129S2-Pak4tm2.1Amin/J) and Albumin-cre mice (B6.Cg-Speer6-ps1Tg(Alb-cre)21Mgn/J). Male and female LKO mice were used at 8 week to 10 week of age.
Wild animals	No wild animals were used in the study.
Reporting on sex	*Indicate if findings apply to only one sex; describe whether sex was considered in study design, methods used for assigning sex. Provide data disaggregated for sex where this information has been collected in the source data as appropriate; provide overall numbers in this Reporting Summary. Please state if this information has not been collected. Report sex-based analyses where performed, justify reasons for lack of sex-based analysis.*
Field-collected samples	No filed-collected samples were used in the study.
Ethics oversight	All animal experiments were performed in accordance with the Guide for the Care and Use of Laboratory Animals published by the US National Institutes of Health (NIH Publication No. 85-23, revised 2011). The study protocol was approved by the Institutional Animal Care and Use Committee of Jeonbuk National University (permit number: JBNU-2019-00122).

Note that full information on the approval of the study protocol must also be provided in the manuscript.
